# The Prevalence of Imposter Syndrome and Its Association with Psychological Distress: A Cross-Sectional Study

**DOI:** 10.1192/j.eurpsy.2026.10650

**Published:** 2026-07-31

**Authors:** A. Al-Lawati, A. J. Al Wahshi, T. Al Mahrouqi, Y. Al Mufargi, S. Al Shukaily, H. Al Aufi, I. Al Shehhi, A. A. Al Azri, H. Al Sinawi

**Affiliations:** 1 College of Medicine and Health Sciences, Sultan Qaboos University; 2 Department of Behavioral Medicine, Sultan Qaboos University Hospital; 3 Department of General Surgery, Medical City Hospital for Military and Security Services; 4 Sultan Qaboos University Hospital, Muscat, Oman

## Abstract

**Introduction:**

Imposter Syndrome (IS) is characterized by persistent self-doubt and feelings of intellectual fraudulence despite evidence of competence. It is highly prevalent among high-achieving individuals, particularly university students. Although IS is believed to be linked with psychological distress such as anxiety and depression, regional data from the Middle East remain limited. Understanding this phenomenon is vital in environments driven by performance.

**Objectives:**

This study aimed to assess the prevalence of IS among undergraduate students at Sultan Qaboos University (SQU) and assess its association with depression and anxiety.

**Methods:**

A cross-sectional study was conducted among 504 stratified-randomly selected full-time undergraduate students at SQU. Participants completed a validated online questionnaire including the Clance Imposter Phenomenon Scale (CIPS), Patient Health Questionnaire-9 (PHQ-9), and Generalized Anxiety Disorder-7 (GAD-7). Statistical analyses included Pearson’s correlation, chi-square testing, and logistic regression.

**Results:**

IS was present in 56% of participants. CIPS scores were moderately correlated with depression (r = 0.486, p < 0.001) and anxiety (r = 0.472, p < 0.001). Students who experienced imposter syndrome showed a higher probability of developing depressive symptoms (χ2 = 45.63, p < 0.001, OR = 3.49) and anxiety symptoms (χ2 = 32.96, p < 0.001, OR = 2.86). Logistic regression showed that depressive (B = 0.096, p < 0.001) and anxiety symptoms (B = 0.075, p = 0.003) were significant predictors of IS.

**Conclusions:**

This study reveals a strong link between imposterism, depression, and anxiety among students, underscoring the need for targeted counseling interventions and clinical recognition of the phenomenon

**Disclosure of Interest:**

None Declared

